# Thalamocortical connectivity is associated with autism symptoms in high-functioning adults with autism and typically developing adults

**DOI:** 10.1038/s41398-021-01221-0

**Published:** 2021-02-03

**Authors:** Rafi Ayub, Kevin L. Sun, Ryan E. Flores, Vicky T. Lam, Booil Jo, Manish Saggar, Lawrence K. Fung

**Affiliations:** 1 Department of Bioengineering, Stanford University, Stanford, CA USA; 2 Department of Psychiatry and Behavioral Sciences, Stanford University, Stanford, CA USA; 3 School of Medicine, Stanford University, Stanford, CA USA

**Keywords:** Autism spectrum disorders, Human behaviour

## Abstract

Alterations in sensorimotor functions are common in individuals with autism spectrum disorder (ASD). Such aberrations suggest the involvement of the thalamus due to its key role in modulating sensorimotor signaling in the cortex. Although previous research has linked atypical thalamocortical connectivity with ASD, investigations of this association in high-functioning adults with autism spectrum disorder (HFASD) are lacking. Here, for the first time, we investigated the resting-state functional connectivity of the thalamus, medial prefrontal, posterior cingulate, and left dorsolateral prefrontal cortices and its association with symptom severity in two matched cohorts of HFASD. The principal cohort consisted of 23 HFASD (mean[SD] 27.1[8.9] years, 39.1% female) and 20 age- and sex-matched typically developing controls (25.1[7.2] years, 30.0% female). The secondary cohort was a subset of the ABIDE database consisting of 58 HFASD (25.4[7.8] years, 37.9% female) and 51 typically developing controls (24.4[6.7] years, 39.2% female). Using seed-based connectivity analysis, between-group differences were revealed as hyperconnectivity in HFASD in the principal cohort between the right thalamus and bilateral precentral/postcentral gyri and between the right thalamus and the right superior parietal lobule. The former was associated with autism-spectrum quotient in a sex-specific manner, and was further validated in the secondary ABIDE cohort. Altogether, we present converging evidence for thalamocortical hyperconnectivity in HFASD that is associated with symptom severity. Our results fill an important knowledge gap regarding atypical thalamocortical connectivity in HFASD, previously only reported in younger cohorts.

## Introduction

Alterations in sensorimotor processing are highly common in individuals with autism spectrum disorder (ASD)^1–4^. Many studies have found evidence for altered auditory processing^5,6^, tactile processing^7,8^, visual processing^9^, and motor learning^10^ in various age groups in ASD. Such sensorimotor differences have been linked to core symptoms of repetitive behaviors and socio-communicative deficits^8,11–15^. Additionally, sensorimotor processing is hypothesized to play a pivotal role in disrupted cognitive functions attributed to ASD, such as information processing, central coherence, perceptual integration, language development, and reading emotions from faces^1,16,17^. This relationship between sensorimotor processing and core symptoms of ASD has been well-studied in younger cohorts. In older cohorts, however, past studies have combined adults with other age groups with limited behavioral and self-report data^18–20^. Even though adults with ASD overwhelmingly experience unusual sensory processing similarly to their younger counterparts^21^, the link between sensorimotor processing and its underlying neurophysiology in adults is poorly explored, highlighting an important clinical need. In the present study, we focused specifically on high-functioning adults with ASD (HFASD) and explored the neural correlates of sensorimotor processing and their relationships with typical characteristics of autism to better inform treatment.

Sensorimotor processing may be related to aberrant inhibitory neurotransmission in ASD. Altered cortical inhibition is a widely supported theory in ASD, as evidenced by altered levels of glutamate^22^ and GABA^23,24^, and sensory hyper- and hyposensitivity^1^. Neurodevelopmental disorders associated with ASD, such as fragile X Syndrome and Tourette syndrome, also exhibit alterations of cortical inhibition and suggest an involvement of sensorimotor deficits^25,26^. Taken together, cortical excitability and sensorimotor processing appear to play an important role in the neurobiology of ASD.

In addition to cortical inhibition, the thalamus has been shown to be associated with the neurobiology of ASD. The thalamus is a critical functional hub for relaying sensory information to the cortex and modulating motor signals^27^. It does so by exerting fine-tuned inhibitory control over cortico-cortical and subcortical-cortical signaling^18,28^. Treatments such as deep brain stimulation targeting the thalamus at the anterior nucleus and the ventral intermediate nucleus are known to reduce seizures in epilepsy^29^ and reduce tremors in Parkinson’s disease^30^, respectively. This inhibitory role of the thalamus in motor processing and the evidence of cortical disinhibition shown in ASD suggest that the connectivity of the thalamus may differ in individuals with ASD.

Resting-state functional magnetic resonance imaging (rsfMRI) has been a widely used tool in assessing co-fluctuations (or functional connectivity; FC) across brain regions in the absence of task-related cognitive demand^31,32^. The task-free nature of rsfMRI has several key benefits, including higher participant compliance and the ability to aggregate data across multiple sites, that enable its widespread use in investigating thalamocortical FC in individuals with ASD. An early neuroimaging study of children and adolescents with ASD using rsfMRI found evidence of thalamocortical hyperconnectivity in limbic, auditory, and motor regions, as well as general hypoconnectivity with regions involved in executive functioning and social cognition^33^. Another investigation that included children, adolescents, and adults from the autism brain imaging data exchange (ABIDE), a multisite neuroimaging database, also revealed widespread thalamic hyperconnectivity with temporal, sensorimotor, and prefrontal cortices with limited evidence of hypoconnectivity. However, these results were primarily driven by adolescents. Importantly, this study’s investigation of the relationship between thalamocortical connectivity and ASD symptoms is inconclusive due to a lack of behavioral data^18^. Other studies that included adults did not use functionally distinct regions-of-interest (ROIs)^19^ and did not examine the differences in thalamocortical connectivity between adults and adolescents^34^, obscuring the neurobiology of thalamocortical connectivity in adults alone. Thus, the results from these combined cohort studies are not representative of HFASD. In fact, no study to date has investigated thalamocortical connectivity specifically in HFASD and its relationship with ASD characteristics.

The overarching goal of this study was to examine thalamocortical FC in the less-studied ASD subpopulation of high functioning adults compared to an age- and sex-matched control group and analyze its relationship with clinical symptoms. We used a generalized linear model (GLM)-based approach for seed-based connectivity analysis and included seeds from the left and right thalami. We additionally used a cortical seed-based approach for analyzing thalamocortical FC^18^ and included seeds from the medial prefrontal cortex (mPFC), posterior cingulate cortex (PCC), and left dorsolateral prefrontal cortex (dlPFC). These seeds allowed us to examine FC in the two most commonly studied resting state networks in ASD, default mode (DMN)^35–37^ and central executive (CEN)^38–42^. These networks have shown altered connectivity with sensorimotor regions in previous studies of adolescents with ASD^37^ and are known to engage the thalamus as a key functional region^27,43^. Then, we examined brain-behavior relationships by correlating observed group differences with clinical markers of ASD. Furthermore, we explored the effects of age and sex on these relationships using mediation and moderation analyses predicting ASD symptom severity. Lastly, we sought to validate our results in a separate, larger cohort of HFASD from the ABIDE public database^44,45^. By studying thalamocortical connectivity in HFASD, we hope to better understand the neural correlates of differential sensorimotor processing in a specific and understudied population.

## Materials and methods

### Participants

Participants were of the same cohort that had been studied in a previous investigation by our lab^24^. We will refer to this cohort as the HFASD cohort. Twenty-eight individuals with ASD and 29 TD controls were recruited. Of these, 26 ASD and 23 TD individuals had completed resting-state fMRI scans. Two TD individuals were removed from this study due to an incomplete IQ assessment or not meeting the inclusion criteria for IQ. An additional three ASD and one TD individuals were excluded due to excess motion in the scanner (see *Supplemental Information*). Thus, in total, twenty-three individuals with ASD (mean[SD] 27.1[8.9] years, 18-48 years; 39.1% female; full-scale IQ 103.7[16.1]) and 20 age- and sex-matched typically developing (25.1[7.2] years, 18-42 years; 30.0% female; full-scale IQ 111.1[14.6]) individuals (Table 1) were included in the present study. Inclusion and exclusion criteria are detailed in Supplemental Table 1 and in Fung et al.^24^.

**Table 1:** HFASD participant demographics and clinical assessments.

	ASD *N* = 23	TD *N* = 20	T-Test / χ^2^ Test *p-value*
Age	27.13 ± 8.92	25.10 ± 7.18	0.4134
Female Sex	39.13%	30.00%	0.5309
Right-handed	84.21%	73.68%	0.4261
FSIQ	103.70 ± 16.09	111.05 ± 14.61	0.1241
VIQ	105.17 ± 19.27	109.65 ± 14.56	0.3919
NVIQ	102.13 ± 13.67	112.40 ± 13.44	**0.01742**
ADOS-2 – Total	10.86 ± 4.87	N/A	N/A
AQ – Total	31.13 ± 6.77	16.65 ± 8.41	**0.00000041**
RAADS-R – Total	124.52 ± 37.20	54.85 ± 44.79	**0.00000297**
SRS-2 – Raw Total	95.04 ± 24.81	44.58 ± 31.71	**0.00000249**

Values reported are mean ± SD unless otherwise noted. Bolded Welch’s T-test/χ^2^ results are *P* < 0.05. FSIQ = Full-scale IQ. VIQ = Verbal IQ. NVIQ = Non-verbal IQ. ADOS-2 = Autism Diagnostic Observation Schedule, Second Edition. AQ = Autism-Spectrum Quotient. RAADS-R = Ritvo Autism-Asperger Diagnostic Scale-Revised. SRS-2=Social Responsiveness Scale, Second Edition.

### Imaging data acquisition

High-resolution 3D T1-weighted structural images were acquired using a 3T GE Signa PET/MR scanner (General Electric, Milwaukee, WI) using the following parameters: repetition time (TR) = 7.74 ms, echo time (TE) = 2.87 ms, flip angle = 12°, acquisition matrix = 256 × 256, inversion time = 450 ms, slice thickness = 1.4 mm, in-plane resolution = 0.94 mm isotropic.

Gradient echo pulse sequence was used for resting-state functional imaging with the following parameters: TR = 2000ms, TE = 30 ms, flip angle = 80°, acquisition matrix = 64 × 64, field of view = 24 cm, slice thickness = 4 mm, and interleaved slices. A fixation cross was displayed throughout the duration of the scan. Subjects were instructed to let their mind wander and encouraged to keep their eyes-open and fixate on the cross while lying in the scanner, promoting a state of wakeful rest. Total scan time was eight minutes for each subject.

### Behavioral assessments

The diagnosis of ASD in participants was confirmed by a combination of Autism Diagnostic Interview-Revised (ADI-R)^46^ and Autism Diagnostic Observation Schedule, Second Edition-2 (ADOS-2)^47^, as well as clinical interview. Verbal and nonverbal IQ was measured using the Stanford-Binet Intelligence Scales, Fifth Edition^48^. All participants completed a series of self-report questionnaires to assess ASD symptoms, including the Ritvo Autism-Asperger Diagnostic Scale-Revised (RAADS-R)^49^, the Social Responsiveness Scale, Second Edition (SRS-2)^50^, and the autism-spectrum quotient (AQ)^51^.

### ABIDE participants

To validate our findings in a separate cohort, we identified 58 ASD and 51 TD individuals from ABIDE matched to the HFASD cohort by age, sex, and IQ (Table 2). The selection process is detailed in *Supplemental Information*. Functional brain imaging data were analyzed with the methods below.

**Table 2:** ABIDE demographics.

	ASD *N* = 58	TD *N* = 51	T-Test / χ^2^ Test *p-value*
Age	25.40 ± 7.81	24.37 ± 6.69	0.4599
Female sex	37.93%	39.22%	0.8906
FSIQ	108.70 ± 14.81	109.31 ± 13.82	0.8229

Values reported are mean ± SD unless otherwise noted. Bolded Welch’s T-test/χ^2^ results are *P* < 0.05. FSIQ = Full-scale IQ.

### fMRI preprocessing

Supplemental Table 2 presents the MRI acquisition parameters for the HFASD and ABIDE cohorts. Functional data from both the HFASD and ABIDE cohorts were preprocessed identically using fMRIPrep v1.3.1^52^, a pipeline that uses a combination of tools from existing neuroimaging software packages.

Nuisance regressors—framewise displacement (FD), average cerebrospinal fluid (CSF) signal, average white matter (WM) signal, and global signal—were extracted using this pipeline. In the HFASD cohort, group-mean FD was 0.162 for ASD and 0.143 for TD (two sample t-test *p* = 0.4029). In the ABIDE cohort, group-mean FD was 0.124 for ASD and 0.123 for TD (two sample t-test *p* = 0.8679). Frames that exhibited an FD of over 0.5 mm were censored from the timeseries, as well as one before and two after the offending frame. In the HFASD cohort, group-mean number of remaining frames out of 240 was 220.96 for ASD and 222.24 for TD (two sample t-test *p* = 0.8954). In the ABIDE cohort, group-mean percentage of frames remaining was 96.25% for ASD and 97.16% for TD (two sample t-test *p* = 0.2806). Participants with <80% of their TRs retained after motion scrubbing were removed from the analysis. As a result, a total of 20 TD participants and 23 ASD participants were analyzed from the HFASD cohort (Table 1). From ABIDE, 51 TD participants and 58 ASD participants were analyzed (Table 2). Further details of the preprocessing are described in *Supplemental Information*.

### Seed-based correlation analysis

The left and right thalami were individually seeded by extracting the average time-series of all voxels within the region defined by the Harvard-Oxford thalamus probabilistic atlas, thresholded at 90%. The mPFC, PCC, and left dlPFC seeds were created using spherical ROIs with 5 mm radii centered at MNI coordinates (0,47,−2), (0,−49,40), and (−38,52,20), respectively. These were the peak coordinates for regions involved in the default mode and executive control networks^53,54^. The resulting time-series from the five ROIs were placed in a first-level GLM to determine the relationship of each seed with every other voxel in the brain. Nuisance regressors were included in the GLM, which consisted of the six rigid body head-motion parameters, CSF, WM, global signal, and their derivatives, resulting in a 18-parameter model. A separate analysis without global signal regression (GSR) was performed to determine if results were influenced by motion artifacts. Frames censored for excessive FD were additionally included in the GLM for motion scrubbing. The first-level GLM analysis was run using FSL FEAT^55^. The resulting parameter estimates for each participant were incorporated in a higher-level GLM, which included any variables with significant group differences as covariates. This higher-level GLM analysis was run using FSL randomise^56^, a permutation-based method that uses threshold-free cluster enhancement to correct for family-wise error rate in order to determine brain areas with group differences in parameter estimates between TD and ASD. Permutation statistical tests were deemed most appropriate for group-level analysis since the distribution of the difference in group means is not known and equal variances were assumed. Finally, for each of these FSL-identified “clusters,” its mean parameter estimate was extracted for each individual using Featquery^55^. The significance threshold for identified clusters was set to *p* = 0.01 after Bonferroni correction for five seeds (α = 0.05, *m* = 5).

To validate our observed connectivity findings in the ABIDE cohort, we ran whole-brain connectivity analysis using a similar GLM approach as in our cohort with the five seeds of interest. To account for potential confounding effects from differences in scan acquisition parameters, we included site as a covariate in our ABIDE GLM analysis as one-hot encoded variables.

### RSFC-behavior correlates in HFASD cohort

Pearson’s correlations were performed between mean parameter estimates for each cluster identified and scores on the behavioral assessments, as the relationship was observed to be linear. Analyses were run within ASD and TD groups separately. Significance was set using the Benjamini-Hochberg false discovery rate-controlling procedure, with level α = 0.05 (2-tailed) and the number of tests *m* = 24 (4 behavioral assessments × 2 groups × 3 RSFC clusters found).

### Post-hoc analyses of age and sex

Post-hoc GLM analyses were used to explore the potential effects of age and sex on group differences in RSFC. ASD/TD group (coded as two binary variables) and age were included as independent variables in one GLM setup; group, age, and a group × age interaction term, in another GLM. Then, we performed the same analyses replacing age with sex. Significance level was set at α = 0.05 (two-tailed).

## Results

### Demographics and behavioral assessments

According to Welch’s t-test and chi-squared test, the ASD and TD groups did not show significant differences in age, sex, and full-scale IQ (Tables 1 and 2). Because mean non-verbal IQ (NVIQ) was identified to be significantly lower in the ASD group than in the TD group in the HFASD cohort, NVIQ was included as a covariate in all subsequent GLM analyses. As expected, the ASD group-mean score was significantly higher than that of TD for AQ, RAADS-R, and SRS-2; these behavioral assessments were identified for use in subsequent RSFC-behavior correlations.

### Thalamic hyperconnectivity in ASD

**Fig. 1:**
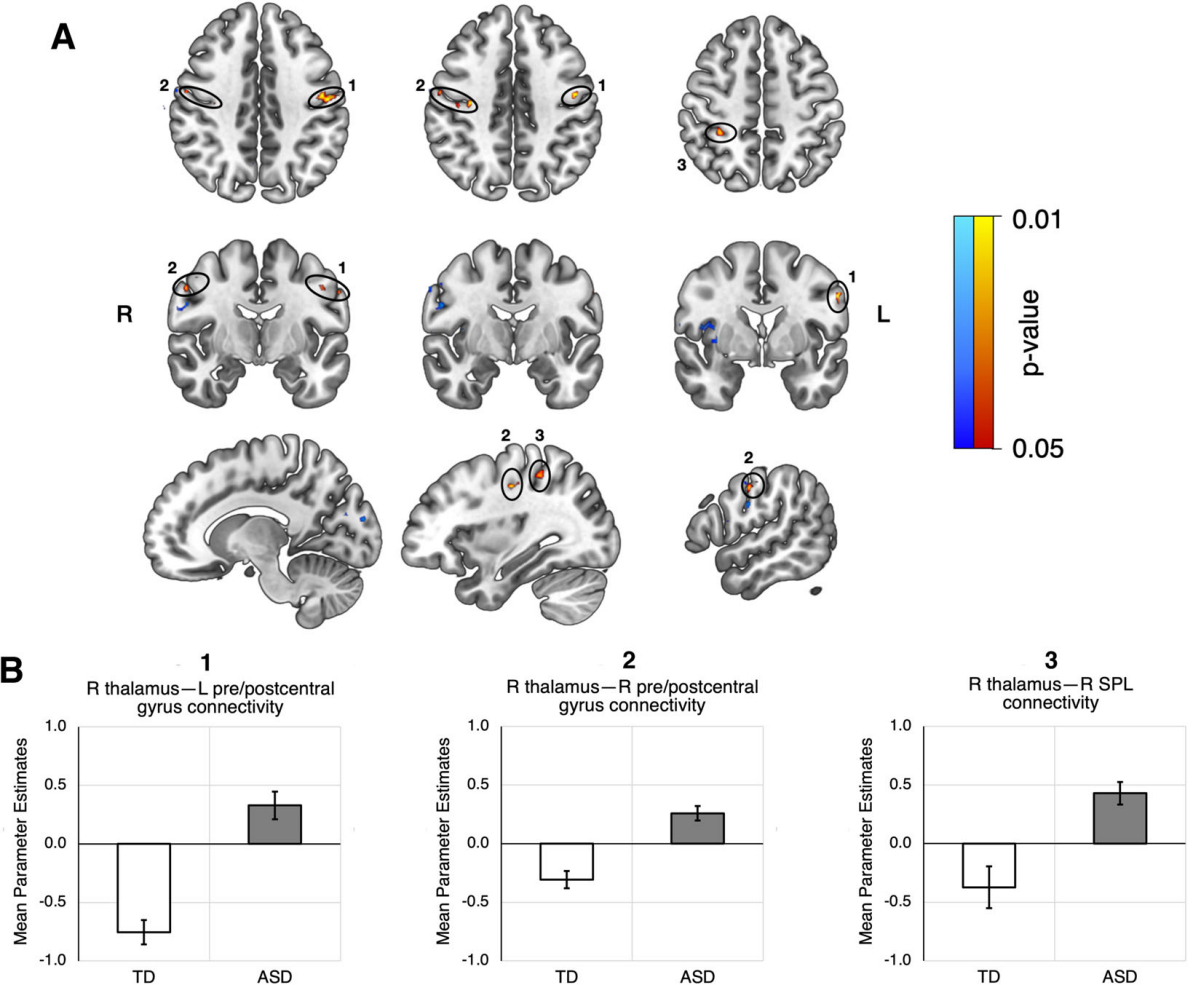
Voxels that were found to exhibit hyperconnectivity with the right thalamus. Significant clusters found in the HFASD (orange) and ABIDE (blue) cohorts are colored by p-value. **A** Top - axial view, middle - coronal view, bottom - sagittal view, sliced from center to right. HFASD clusters are primarily localized in the left and right precentral gyri, left and right postcentral gyri, and right superior parietal lobule. ABIDE clusters are primarily localized in the right precentral and postcentral gyri, right insulae, and right and left cuneus. **B** Bar graphs depicting group differences in group-level GLM parameter estimates, taken as an average of all the voxels in a particular cluster for each subject. The two clusters in the right precentral and postcentral gyri (Table 3) were averaged together as one cluster. SPL = superior parietal lobule.

In our seed-based correlation analysis, the GLM for the right thalamus seed identified several clusters that indicated hyperconnectivity in the ASD group (Table 3). The largest of these were found in the left precentral and postcentral gyri (*p* = 0.007), right precentral and postcentral gyri (*p* = 0.01), and right superior parietal lobule (*p* = 0.012) (Fig. 1). After Bonferroni correction for five seeds, only the bilateral precentral and postcentral gyri retained a significant difference in mean parameter estimates between ASD and TD groups; the right superior parietal lobule was trending towards significance (Fig. 1B). No significant clusters were found for the left thalamus, mPFC, dlPFC, and PCC seeds.

**Table 3:** Significant clusters found in seed connectivity analysis.

Contrast	Anatomical region	Voxels	p-value	Max X (MNI)	Max Y (MNI)	Max Z (MNI)
HFASD R thalamus, ASD > TD	L precentral gyrus, L postcentral gyrus	107	0.007	−58.5	−2.5	31.5
	R precentral gyrus, R postcentral gyrus	44	0.01	31.5	−16.5	41.5
	R superior parietal lobule, R postcentral gyrus	33	0.012	31.5	−40.5	51.5
	R precentral gyrus, R postcentral gyrus	25	0.012	55.5	−8.5	41.5
ABIDE R thalamus, ASD > TD	R insula, R central operculum	75	0.024	36	6	10
	R precentral gyrus, R postcentral gyrus	34	0.019	54	−6	28
	R cuneus	17	0.027	8	−68	20
	L cuneus	16	0.031	−12	−80	20
	R cuneus, L cuneus	16	0.036	2	−74	20
	R precentral gyrus, R pars opercularis	15	0.028	50	4	18

Clusters with less than 15 voxels were not included.

In post-hoc GLM analyses, age and sex did not mediate or moderate the group difference in connectivity. Additionally, when global signal regression was not performed in the seed-based correlation analysis, we did not observe any significant clusters for the right thalamus seed.

### RSFC–behavior correlates

**Fig. 2:**
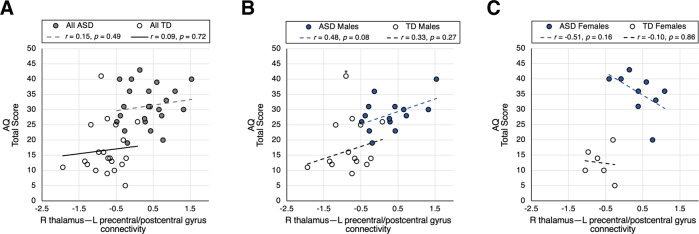
Correlations of left precentral/postcentral gyri cluster with AQ. Correlations by group (**A**), in males by group (**B**), and in females by group (**C**). Reported *r* values are Pearson’s correlation coefficients.

In neither the ASD group nor the TD group were significant correlations found between behavioral assessment scores and mean parameter estimates of the identified clusters. As shown in Fig. 2A and Supplemental Fig. 1, within-group Pearson correlation coefficients between AQ, RAADS-R, or SRS-2 raw total scores and right thalamus–left precentral/postcentral gyrus connectivity never exceeded 0.3.

In an exploratory fashion, we looked into sex as a possible complicating factor of group-wise analyses. Relationships between right thalamus–left precentral/postcentral gyrus RSFC and ASD symptom severity were found to differ by sex (Fig. 2B, C). In ASD males, a positive linear relationship approaching significance was noted between AQ total score and right thalamus–left precentral/postcentral gyrus RSFC (*r* = 0.48, *p* = 0.079). In ASD females, a negative linear relationship was found, though the data were too limited to reach significance (*r* = –0.51, *p* = 0.16).

To follow up on these findings, we performed a moderation analysis of the ASD group to investigate whether sex moderates the effect of RSFC on behavior. We included sex, RSFC, and a sex × RSFC interaction term as independent variables of a GLM predicting AQ total score. Significance was set at *p* < 0.05. Sex was found to be an effect modifier of the relationship between AQ and RSFC (model *F*(3,19) = 5.059, *p* = 0.00962, adjusted *R*^2^ = 0.3563; sex × RSFC interaction *p* = 0.0238).

Further GLM analyses of the HFASD cohort were performed to explore sex differences in the relationship between other ASD behavioral measures and right thalamus–left precentral/postcentral gyrus RSFC. These analyses can be found in the *Supplemental Information*.

### ABIDE validation

In the ABIDE GLM analysis, clusters were found that had greater connectivity with the right thalamus in the ASD group compared to the TD group. Two of these clusters were located in the right precentral (*p* = 0.019) and postcentral gyri (*p* = 0.028), validating the observed hyperconnectivity of the same regions in our HFASD cohort. The other clusters were primarily localized in the insula and the cuneus (Table 3) and not in areas originally found in our cohort. The parameter estimates for the largest cluster identified also did not significantly correlate with any clinical measures provided in ABIDE.

## Discussion

The goal of this study was to investigate thalamocortical connectivity in HFASD and how it relates to ASD symptom severity. We performed seed-connectivity analysis investigating the default mode, executive control, and thalamocortical networks in an age- and sex-matched cohort of HFASD and their typically developing counterparts. We observed hyperconnectivity of the right thalamus with bilateral pre- and post-central gyri in the ASD group, but no other seeded regions had significant group differences in our HFASD cohort. We further validated right thalamocortical hyperconnectivity with right precentral and postcentral gyri in a similar cohort of HFASD in ABIDE. Taken together, these results provide support for the presence of hyperconnectivity of the right thalamus to sensorimotor regions in the cortex in HFASD.

The lack of group differences for seeded regions in the DMN and CEN is surprising given the widespread evidence for altered connectivity in these networks across age groups^31,33,35,37,57^. One study of male high-functioning ASD adults found hypoconnectivity between core DMN regions that negatively correlated with AQ^36^. However, our cohort consists of both males and females, and sex is known to mediate DMN connectivity^58^. CEN connectivity in autism has mixed results. Studies of ASD children and adolescents show hypoconnectivity with prefrontal areas^33,57^, but a large scale investigation of ABIDE found no group differences in connectivity in prefrontal areas in only adults^18^, corroborating our results. Sex may have also influenced our CEN results, but it is difficult to draw conclusions with our modest sample size. Further investigation into sex-mediated effects on DMN and CEN connectivity in high-functioning ASD adults is needed.

The differences in connectivity from the thalamus to the pre- and post-central gyri form a strong case for impairment of sensorimotor processing in HFASD. Undoubtedly, the pre- and post-central gyri are critical for the execution of motor commands and perception of sensory stimuli, respectively. One study has previously shown that the connectivity of the thalamus to bilateral pre- and post-central gyrus and the left superior parietal lobule is disrupted in ASD adolescents in response to aversive auditory and tactile stimuli^59^. Indeed, atypical thalamic connectivity to sensorimotor cortical regions is widely replicated in studies of children and adolescents with ASD^18,19,33,34,57^. Thus, our results demonstrate the presence of atypical thalamocortical connectivity to sensorimotor regions in HFASD and reinforce the notion that atypical thalamocortical connectivity is a neural correlate of sensorimotor processing differences in ASD.

While the link between sensory abnormalities and thalamocortical dysconnectivity seems to be clear, the effects of age on this relationship in ASD are unclear. Our post-hoc GLM analyses generally showed no significant main effect or interaction effect due to age. Hyperconnectivity between the thalami and sensorimotor regions was demonstrated in children and adolescents with ASD in several recent reports^18–20,33,57^, consistent with our finding in HFASD in this study. To fully understand the effects of age on thalamocortical dysconnectivity, we will need to perform further investigations using a longitudinal design.

In addition to age, sex is known to drive considerable variation in the clinical presentation of ASD^60^. Acknowledging our modest sample size, we explored potential sex differences in our observed brain-behavior relations and report preliminary observations (see Fig. 2B, C and *Supplemental Information*). We found that ASD males exhibited a positive correlation between sensorimotor thalamocortical connectivity and AQ, while ASD females exhibited a negative correlation. Furthermore, for males but not females, the thalamocortical connectivity partially mediated the ASD vs. TD group difference in AQ. This suggests that sensorimotor function may affect ASD characteristics differentially by sex in adults with ASD. In fact, a recent meta-analysis revealed that high-functioning female adults with ASD typically report disproportionately more sensorimotor symptoms than their ASD male counterparts^61^. A potential mechanism of sex differences in sensorimotor processing could be differences in inhibitory neurotransmission. In our previous study of GABA concentrations in HFASD, we observed that ASD males had a negative correlation of thalamic GABA concentration with AQ, but ASD females had a positive correlation, a similar sex-specific relationship as observed in the present study^24^. Altogether, these preliminary results, in conjunction with the findings from past studies, demonstrate the impact of sex on ASD neurobiology and underscore the need for investigating sex differences in studies of ASD.

The presence of thalamocortical hyperconnectivity further suggests awry inhibitory mechanisms. Inhibitory neurons in the thalamus are known to counter the synchronizing tendency of excitatory thalamocortical neurons, mediated by GABA_A_ receptors^62^. In fact, our earlier study on the same cohort using proton magnetic resonance spectroscopy revealed decreased thalamic concentrations of GABA in ASD males compared to their TD counterparts^24^. Additionally, GABA has previously been associated with the integrity of synchronized oscillations in sensory processing^16^. While hyperconnectivity may indicate altered function in the GABAergic system in the thalamus, biophysical modeling is needed to explore the mechanisms of how thalamic inhibition can impact thalamocortical functional connectivity^63^.

Our results indicate group differences in functional connectivity of the right thalamus but not the left. Similar asymmetries have been found previously in a study of children with ASD, which observed greater white matter compromise, greater thalamic functional specialization, and maladaptive temporo-thalamic hyperconnectivity in the right hemisphere^57^. To rule out the possibility of a mediating effect of handedness, we ensured that the proportion of individuals with ASD who were right-handed did not differ significantly from that of controls (Table 1). Other explanations for this asymmetry may be found through other studies of the thalamus in ASD. One study investigating serotonin levels in thalamic pathways finds asymmetries in children with ASD in serotonin synthesis in the thalamus, frontal cortex, and dentate gyrus^64^. Interestingly, serotonin has been previously implicated in the hyperexcitability of sensory thalamocortical circuits^65^. Thus, the asymmetry of thalamic hyperconnectivity with the sensorimotor cortex further suggests an underlying disruption of neurotransmitter systems in the thalamus.

There are several limitations to the present study. First, the modest size of the investigated cohort limits the generalizability of our results. Drawing conclusions from the observed brain-behavior relationships with a small sample size is difficult given the heterogeneities already present in the ASD population. Second, there is a significant group difference in NVIQ between the HFASD and TD groups. Moreover, our GLM analyses that accounted for NVIQ as a covariate revealed that the results are not affected by between-group and within-group variation in NVIQ. This suggests that sensorimotor hyperconnectivity with the thalamus and its relation to core behavioral abnormalities in ASD is independent of NVIQ. Third, the observed lack of group differences in connectivity without GSR may diminish the robustness of our findings. However, our results are highly unlikely to be contaminated by motion artifacts; GSR is known to be effective at removing spurious low-amplitude correlations introduced by head motion^66,67^, and overall head motion was comparable between the ASD and TD groups. Even though the inclusion of GSR in the preprocessing of resting-state fMRI is still highly contended^68^, it is especially necessary for removing motion artifacts in studies of ASD, a population that tends towards excessive head motion^69^. One study even found GSR boosted variance explained by whole-brain RSFC in behavioral measures, though it is unclear if the same applies for specific connections as in our study^70^. For these reasons, GSR is warranted. Fourth, the seed-connectivity analysis in the present study used whole-thalamus seeds instead of individual thalamic nuclei. Both the HFASD and ABIDE cohorts used low-resolution functional imaging data, thus results of seed-connectivity analysis using individual thalamic nuclei would have been highly susceptible to noise. Additionally, most investigations of thalamocortical connectivity have used a hybrid whole-thalamus and cortical ROI seed approach^18^, which we follow. Future investigations ought to examine the connectivity of specific thalamic nuclei to further elucidate the relationship between thalamocortical connectivity and sensorimotor function in ASD. Fifth, the lack of measures for sensorimotor symptoms could limit the extension of our conclusions to impaired sensorimotor processing in ASD. In future studies, we will prioritize the characterization of sensorimotor symptoms accordingly. Lastly, the use of only self-report measures may be problematic due to the subjectivity of reporting symptom severity.

Despite these limitations, this study reports findings of resting-state thalamocortical functional hyperconnectivity with the sensorimotor regions in HFASD for the first time. This hyperconnectivity is associated with autistic features across all participants. These results confirm existing literature on the widespread hyperconnectivity of the thalamus with the cortex and strongly suggest the presence of differences in sensorimotor processing in ASD. These differences could be indicative of disrupted neurotransmitter systems in the thalamus and can be attributed to the neurobiology of ASD independent of typically associated cognitive differences and age. Further investigation into the underlying mechanisms of disrupted thalamocortical connectivity and how it may lead to sex-specific abnormal sensory processing is encouraged.

## Supplementary information

Supplemental Material
