# Supplementary material for: Thalamocortical connectivity is associated with autism symptoms in high-functioning adults with autism and typically developing adults
Source: Transl Psychiatry. 2021 Feb 3;11:93. doi: 10.1038/s41398-021-01221-0 (PMC7859407; doi:10.1038/s41398-021-01221-0)
Supplement: Supplementary file 1 — Supplemental Material [file 41398_2021_1221_MOESM1_ESM.docx]

**Supplemental Information**

*Selection of ABIDE subjects*

ABIDE I and ABIDE II include data from a total of 2226 participants. First, we removed 1603 ASD and TD participants who were younger than 18 or older than 55. Then, we removed 72 ASD and TD participants whose full-scale IQs were lower than 70. Of the remaining 551 participants, only 29 were females with ASD; all of these were included in the fMRI analysis, with 22 remaining after removing participants for motion (see main text, Materials and Methods: *fMRI preprocessing*). 36 males with ASD were chosen to achieve similar sex balance in the ABIDE ASD cohort as in our HFASD cohort (39.3% female). Furthermore, they were chosen so that the entire ABIDE ASD cohort would be age- and IQ-matched as closely as possible with our HFASD cohort (Table 1). After the ABIDE ASD cohort was selected, 51 ABIDE TD individuals were chosen to achieve similar ASD/TD group balance as in our HFASD cohort (53.5% ASD), and to be age-, sex-, and IQ-matched with the ABIDE ASD cohort. The resulting ABIDE cohort included 58 individuals with ASD (mean[SD] 25.4[7.8] years; 37.9% female; IQ 108.7[14.8]) and 51 age-, sex- and IQ-matched TD individuals (24.4[6.7] years; 39.2% female; IQ 109.3[13.8]) (Table 2). Behavioral measures from clinical assessments were largely unavailable for this resulting ABIDE cohort.

Of the 109 individuals, 32 were selected from ABIDE I, with scans from the California Institute of Technology (6 participants), Carnegie Mellon University (2), University of Leuven (2), Ludwig Maximilians University Munich (5), NYU Langone Medical Center (7), University of Pittsburgh School of Medicine (1), Trinity Centre for Health Sciences (1), University of Michigan (1), and University of Utah School of Medicine (7). From ABIDE II, individuals were chosen from the Barrow Neurological Institute (7), ETH Zürich (2), Institut Pasteur and Robert Debré Hospital (1), Indiana University (7), Katholieke Universiteit Leuven (26), NYU Langone Medical Center (2), Olin Neuropsychiatry Research Center, Institute of Living at Hartford Hospital (14), San Diego State University (1), Trinity Centre for Health Sciences (9), and University of Utah School of Medicine (8).

*fMRI preprocessing*

For each participant’s functional imaging data, a reference volume and its skull-stripped version were first generated. This was then co-registered to the participant’s T1-weighted image using FSL’s flirt [1] with boundary-based registration [2] using nine degrees of freedom. Six head-motion parameters were estimated using FSL’s mcflirt [3] prior to any spatiotemporal filtering, followed by slice-timing correction using 3dTshift from AFNI [4]. Afterwards, the BOLD time-series data were normalized to MNI152 space (2mm isotropic) and spatially smoothed with an isotropic Gaussian kernel of 6mm full-width half-maximum.

*Mediation analysis stratified by sex*

With all HFASD participants, after stratifying by sex, we performed mediation analysis using the method of Baron and Kenny [5]. The temporal ordering between RSFC and behavior being unclear, we used two analyses. To assess RSFC’s mediation of main effect of group on behavioral score, we included group and RSFC as independent variables of a GLM predicting behavioral score. To assess behavior’s mediation of main effect of group on RSFC, we included group and behavioral score as independent variables of a GLM predicting RSFC. Significance was set at p < 0.05.

First, we investigated males. We removed a TD outlier with AQ score of 41 (mean[SD] = 18.43[9.02] for TD males, including the outlier). GLM mediation analysis found that RSFC was a partial mediator of group difference in AQ (model *F*(2,24) = 17.41, p = 2.134 × 10^-5^, adjusted R^2^ = 0.5579; RSFC term p = 0.0419; group term p = 0.0200, group ∆|ß| = –37.3%). Conversely, AQ score was also found to mediate group difference in RSFC (model *F*(2,24) = 15.34, p = 5.115 × 10^-5^, adjusted R^2^ = 0.5245; AQ term p = 0.0419; group term p = 0.0546, group ∆|ß| = –43.2%).

We then investigated females. RSFC did not mediate the effect of group on AQ (model *F*(2,12) = 27.42, p = 3.348 × 10^-5^, adjusted R^2^ = 0.7905; RSFC term p = 0.1471; group term p = 0.000108, group ∆|ß| = +27.8%). In addition, AQ did not mediate the effect of group on RSFC (model *F*(2,12) = 13.37, p = 8.821 × 10^-4^, adjusted R^2^ = 0.6387; AQ term p = 0.1471; group term p = 0.00325, group ∆|ß| = +59.9%).

*Moderation analysis in ASD*

In the ASD group of the HFASD cohort, we performed moderation analyses to investigate whether sex moderates the effect of RSFC on behavior. We included sex, RSFC, and a sex × RSFC interaction term as independent variables of a GLM predicting behavioral score. This was performed for other behavioral assessments besides AQ: RAADS-R total score and SRS-2 raw total score (Supplemental Figure 1B,C,E,F). Significance was set at p < 0.05. Trends in the data were similar to those for AQ, but sex was not found to be a significant effect modifier of RSFC’s relationships with these other measures.

*Limitations*

These results should be interpreted with caution, since we have few participants to separate by sex, and since the temporal order of behavior and RSFC remains unclear after our analyses. However, at the very least, they warrant further investigation into sex differences in brain-behavior relationships in ASD.

**Supplemental Table 1. Inclusion and exclusion criteria for HFASD cohort.** All female participants were scanned in the follicular phase, estimated from the participants’ history of menses, to minimize possible confounding effects of progesterone.

|  | ASD | TD |
| --- | --- | --- |
| Inclusion | - Diagnosis of ASD based on DSM-5 criteria as confirmed by a qualified clinician, and the administration of Autism Diagnostic Interview-Revised (ADI-R) and Autism Diagnostic Observation Schedule, Second Edition-2 (ADOS-2) - Age 18 to 55 - Post-pubertal adults who are physically healthy - No significant current psychosocial stressors per history - Full scale IQ ≥70 | - Age 18 to 55 - Post-pubertal adults who are physically healthy - No significant current psychosocial stressors per history - Full scale IQ ≥70 |
| Exclusion | - Pre-term birth (<34 weeks’ gestation) - Low birth weight (<2000g) - DSM-5 diagnosis of other severe psychiatric disorder such as bipolar disorder or schizophrenia - History of alcoholism or current substance abuse - Active medical problems such as unstable seizures, congenital heart disease, endocrine disorders - Significant sensory impairments such as blindness or deafness - Contraindication for MRI - Pregnancy - Evidence of any genetic syndrome | - Pre-term birth (<34 weeks’ gestation) - Low birth weight (<2000g) - DSM-5 diagnosis of other severe psychiatric disorder such as bipolar disorder or schizophrenia - History of alcoholism or current substance abuse - Active medical problems such as unstable seizures, congenital heart disease, endocrine disorders - Significant sensory impairments such as blindness or deafness - Contraindication for MRI - Pregnancy - Evidence of any genetic syndrome - Current or past neurological disorders - Current or past psychiatric disorders on the basis of clinical psychiatric evaluation - History of significant perinatal difficulties or abnormal developmental milestones |

**Supplemental Table 2.** **MRI acquisition parameters for HFASD and ABIDE cohorts.**

|  |  | *TR (ms)* | *TE (ms)* | *Flip angle (degrees)* | *Slice acquisition* | *Voxel size (mm)* |
| --- | --- | --- | --- | --- | --- | --- |
| **HFA** | | 2000 | 30 | 80 | interleaved | 3.75×3.75×4.00 |
| **ABIDE** | *Caltech* | 2000 | 30 | 75 | interleaved | 3.5×3.5×3.5 |
|  | *CMU* | 2000/1500 | 30 | 73 | interleaved | 3.0×3.0×4.5 |
|  | *Leuven* | 1666 | 33 | 90 | ascending | 3.594×3.594×4.000 |
|  | *LMU* | 3000 | 30 | 80 | interleaved | 3.0×3.0×4.4 |
|  | *NYU* | 2000 | 15 | 90 | interleaved | 3×3×4 |
|  | *Pitt* | 1500 | 25 | 70 | interleaved | 3.125×3.125×4.000 |
|  | *Trinity* | 2000 | 28 | 90 | ascending | 3.00×3.00×3.84 |
|  | *Michigan* | 2000 | 30 | 90 | ascending | 3.44×3.44×3.00 |
|  | *Utah* | 2000 | 28 | 90 | interleaved | 3.44×3.44×3.3 |
|  | *BNI* | 3000 | 25 | 80 | ascending | 3.75×3.75×4.00 |
|  | *ETH Zurich* | 2000 | 25 | 90 | descending | 3.0×3.0×3.3 |
|  | *IP* | 2700 | 45 | 90 | interleaved | 3.59×3.59×4.00 |
|  | *IU* | 813 | 28 | 60 | interleaved | 3.44×3.44×3.4 |
|  | *KUL* | 2500 | 30 | 90 | ascending | 1.56×1.56×3.1 |
|  | *OLIN* | 1500 | 27 | 60 | ascending | 3×3×3 |
|  | *SDSU* | 2000 | 30 | 90 | interleaved | 3.44×3.44×3.40 |

**Supplemental Figure 1.** **Correlations of left precentral/postcentral gyri cluster parameter estimates with behavioral scores, stratified by sex**. Correlation with RAADS-R Total Score, all participants (A), males (B) and females (C); SRS Raw Total Score, all participants (D), males (E) and females (F) Asterisks denote outliers that were removed for the calculation of simple correlation coefficients *r* for each trendline.

**References**

1. Jenkinson M, Smith S. A global optimisation method for robust affine registration of brain images. Med Image Anal. 2001;5:143–156.

2. Greve DN, Fischl B. Accurate and robust brain image alignment using boundary-based registration. Neuroimage. 2009;48:63–72.

3. Jenkinson M, Bannister P, Brady M, Smith S. Improved Optimization for the Robust and Accurate Linear Registration and Motion Correction of Brain Images. Neuroimage. 2002;17:825–841.

4. Cox RW, Hyde JS. Software tools for analysis and visualization of fMRI data. NMR Biomed. 1997;10:171–178.

5. Baron RM, Kenny DA. The moderator–mediator variable distinction in social psychological research: Conceptual, strategic, and statistical considerations. J Pers Soc Psychol. 1986;51:1173–1182.
